# Use of Ultrasound for Evaluation of a Large Undifferentiated Pleomorphic Sarcoma in the Lower Extremity of an Elderly Male

**DOI:** 10.7759/cureus.30153

**Published:** 2022-10-10

**Authors:** Pasquale Gencarelli, Ashna Hali, Jesper Aurup

**Affiliations:** 1 Department of Emergency Medicine, Rutgers Robert Wood Johnson Medical School, New Brunswick, USA

**Keywords:** soft tissue neoplasm, sarcoma, malignant fibrous histiocytic sarcoma, malignant fibrous histiocytoma, undifferentiated pleomorphic sarcoma

## Abstract

Soft tissue sarcomas arise infrequently and make up less than 1% of all cancers. An undifferentiated pleomorphic sarcoma (UPS), formerly known as a malignant fibrous histiocytoma, is a malignant subtype of soft tissue sarcoma identified by a lack of specific immunohistochemical markers for lineage differentiation. These tumors are aggressive and enlarge rapidly with an increased risk of metastasis, thus prompt diagnosis, treatment, and post-resection surveillance are imperative. This case report demonstrates an incidental finding of a large undifferentiated pleomorphic sarcoma of the posterior thigh in an 86-year-old male initially evaluated with a bedside ultrasound.

## Introduction

Soft tissue sarcomas arise infrequently and make up less than 1% of all cancers. An undifferentiated pleomorphic sarcoma (UPS), formerly known as a malignant fibrous histiocytoma and regrouped by the World Health Organization in 2002, is a malignant subtype of soft tissue sarcoma [1]. UPS is one of the five most common soft tissue sarcomas and most commonly involves the extremities, trunk, and retroperitoneum, and less commonly the head and neck [2,3]. The neoplasm typically presents in the latter decades of life with a slight male predominance [4]. UPS has been classified into five subtypes, including storiform/pleomorphic, myxoid, giant cell, inflammatory, and angiomatoid; the most common subtype is storiform/pleomorphic, forming nearly half of all UPS [5].

Risk factors for UPS include previous radiotherapy and older age [6]. Moreover, the sites of metastasis of UPS most frequently include the lungs or lymph nodes [4,7]. Lastly, computed tomography (CT) or magnetic resonance imaging (MRI), as well as biopsy, are utilized to make the diagnosis, with surgical resection as the main treatment modality. The role of adjuvant/neoadjuvant chemotherapy and radiotherapy have not been clearly defined, but have been utilized for patients with metastasis [8]. We present a case of a large UPS in the right hamstring of a healthy 86-year-old male, initially evaluated with a bedside ultrasound and treated with surgical resection. We performed a review of the literature demonstrating this is one of the largest UPS that has been reported and successfully resected [8-10].

## Case presentation

**Figure 1:**
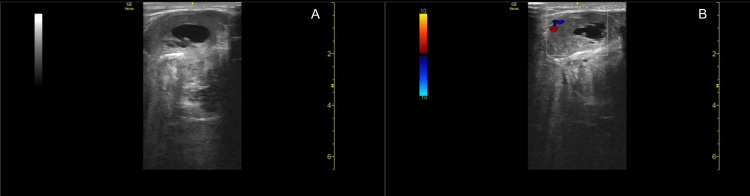
Bedside ultrasound. Two views of the initial bedside ultrasound of the right posterior thigh demonstrating a heterogeneous mass with a hypoechoic center (A) with questionable vascular bundle invasion on the Doppler setting (B).

The patient is an 86-year-old male with no significant past medical history who initially presented to the emergency department for pain and swelling of the right posterior thigh for the past one week. Family members noted the patient has had the mass for years, but over the past few days it had increased in size, and therefore, the patient was brought in to be evaluated in the emergency department. The patient’s physical examination was unremarkable except for moderate swelling and tenderness of the right posterior thigh with no distal erythema or palpable cord. In the emergency department, a bedside ultrasound (Figure 1) of the right posterior thigh demonstrated a heterogeneous mass with concern for necrotic tumor versus abscess given the hypoechoic center of the lesion. The patient’s complete blood count and basic metabolic panel were unremarkable except for an elevated white blood cell count of 17.3 K/uL (range 4.0-10.0 K/uL); however, the patient had no systemic signs of infection with a temperature of 97.4 F oral, pulse of 87 beats per minute, respirations of 16 breaths per minute, blood pressure of 136/62 mmHg, and pulse oximetry of 99% on room air. 

**Figure 2:**
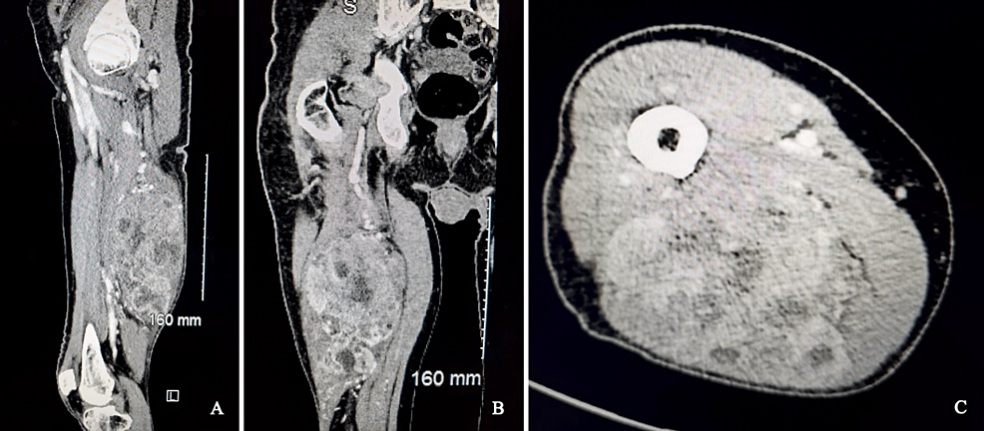
Computed tomography (CT) images. Sagittal (A), coronal (B), and axial (C) CT images of the right lower extremity with intravenous contrast demonstrate a 16.0 × 8.1 × 9.5 cm heterogeneously enhancing mass in the right hamstring muscle.

Subsequent CT imaging of the right lower extremity with IV contrast (Figure 2) revealed a 16.0 × 8.1 × 9.5 cm heterogeneously enhancing mass in the right hamstring muscle, highly suspicious for a malignant lesion. The surgical team was consulted, and after evaluation of the patient in the emergency department, the team recommended discharge with immediate outpatient follow-up with a surgical oncologist. The patient proceeded to follow-up as an outpatient and underwent an incisional biopsy of the lesion by an experienced surgeon. Immunohistochemistry demonstrated positive staining for fibrohistiocytic marker CD 163 and was negative for S100 as well as smooth muscle (actin) and skeletal muscle (desmin) differentiation. The immunohistochemical staining together with the morphology findings was consistent with a high-grade UPS. The patient was scheduled for radical resection of the UPS with adductor magnus muscle transposition a few days after the biopsy diagnosis. Following surgery, the patient was extubated in the post-anesthesia care unit and had no intra- or postoperative complications. The patient received no neoadjuvant/adjuvant chemotherapy or radiation as the lesion did not involve the neurovascular bundle or have osseous involvement. The patient’s metastatic workup, including a CT scan of the chest, was also negative. After nine months of follow-up, the patient ultimately elected to stop further treatment and has since been lost to follow-up.

## Discussion

UPS, also known as malignant fibrous histiocytoma, is classified as a high-grade pleomorphic malignant tumor that lacks specific immunohistochemical markers for lineage differentiation [11]. UPS is one of the five most common soft tissue sarcomas, and most commonly involves the extremities, trunk, and retroperitoneum [2,3]. Moreover, the neoplasm typically presents in the latter decades of life with a slight male predominance [4]. As such, all patients diagnosed with UPS should have CT imaging of the chest to evaluate for pulmonary metastasis. 

The standard of care for UPS includes an en bloc surgical resection with microscopically negative margins followed by serial monitoring by CT or MRI for surveillance of recurrence or late pulmonary metastasis [12]. The role of adjuvant/neoadjuvant chemotherapy and radiotherapy have not been clearly defined, but have been utilized for patients with metastasis [8]. Early disease recognition with complete resection margins is the most important prognostic factor for long-term survival, with distant metastases and local recurrence being associated with higher mortality [8,12]. A retrospective study of 319 patients by Winchester et al. noted recurrence risk was significantly increased with preoperative tumors larger than 5 cm in diameter, invasion beyond the subcutaneous fat, and advanced American Joint Committee on Cancer (AJCC) staging at initial presentation [13]. Metastasis risk was significantly increased in tumors greater than 2 cm and those with lymphatic or vascular invasion, with a propensity to metastasize to the lungs [4,7,13]. Fortunately, our patient had a negative metastatic workup including a CT scan of the chest and the UPS did not involve the neurovascular bundle or osseous structures of the right lower limb.

UPS is rare and delay to diagnosis is common as the neoplasms are not typically included in the physician’s differential diagnosis. Moreover, because UPS usually presents as asymptomatic, rapidly growing cutaneous or subcutaneous nodules without superficial skin abnormalities, patients often do not seek immediate care [14]. Our patient’s disease course underscores the indolent nature of the tumor, highlighted by the large size of the tumor, 16.0 × 8.1 × 9.5 cm, at the time of presentation. Therefore, it is imperative to refer patients with suspected soft tissue sarcomas to specialized centers to establish the diagnosis as the prognosis improves substantially with early diagnosis at a smaller size [12,15].

Furthermore, the diagnostic evaluation of UPS varies depending on the location of the tumor. MRI is recommended for initial imaging of soft tissue masses in the head/neck, trunk, and extremities, while CT is recommended for imaging of retroperitoneal and visceral masses [12]. However, while MRI is the gold standard for the diagnosis of UPS in the extremities, we would like to highlight the benefit of utilizing bedside ultrasound as a fast, non-invasive, and inexpensive diagnostic test. The use of bedside ultrasound in the emergency department allowed providers to efficiently guide the course of treatment for this patient, minimizing the need for additional diagnostic tests or consultations before narrowing the differential. Thus, we advocate for the frequent use of bedside ultrasound in the emergency department to aid providers in establishing a diagnosis. We feel that ultrasound can be incorporated before the use of MRI when evaluating masses of the extremities to decrease the use of unnecessary and expensive imaging should ultrasound findings mitigate concern for a malignant lesion.

To date, evidence is lacking to recommend sentinel lymph node biopsy or positron emission tomography for the initial staging of soft tissue sarcoma. Due to its high diagnostic accuracy, a core needle biopsy is preferred over an open incisional biopsy for the initial evaluation of UPS. The latter should be performed by an experienced surgeon considering the future resection incision, in the case of our patient, and to be sure to minimize dissection and hemorrhage risk [12]. Due to insufficient sampling, fine-needle aspiration is not recommended. Lastly, the need for a multidisciplinary team is critical for the management of patients with musculoskeletal lesions, such as UPS [16]. Abboud et al. demonstrated that multidisciplinary tumor board evaluation of musculoskeletal lesions resulted in a lower repeat image-guided biopsy rate and improved clinical utility of repeat image-guided biopsy species compared to initial nondiagnostic image-guided biopsies [16]. The authors also emphasized the importance of MRI when determining repeat image-guided biopsy trajectory [16].

## Conclusions

Soft tissue sarcomas arise infrequently and make up less than 1% of all cancers; however, they should be included in the differential diagnosis when a patient presents with a rapidly enlarging, painless mass in the head/neck, trunk, extremities, or retroperitoneum. Sarcomas enlarge rapidly, as evident by our patient with a 16.0 × 8.1 × 9.5 cm tumor at presentation. It is imperative to prioritize prompt diagnosis as the risk of metastasis increases as the tumor grows in size and/or invades local structures; bedside ultrasound may serve as a fast, non-invasive, and inexpensive initial diagnostic test. MRI and CT are currently the preferred imaging choices, in conjunction with core needle biopsy, to establish the diagnosis. At present, standard treatment consists of en bloc surgical resection with microscopically negative margins. Serial surveillance with MRI or CT should be conducted after surgical resection to monitor for recurrence or metastasis.
